# Brooklyn Hahnemannian Union

**Published:** 1897-03

**Authors:** 


### BROOKLYN HAHNEMANNIAN UNION.

On Saturday evening, January 30th, members of the Brooklyn Hahnemannian Union celebrated the first anniversary of their existence as a society. The meeting was held, as usual, at the residence of Dr. Close, 64 Willoughby Avenue. Every member was present except Dr. Baylies, who had another engagement, and Dr. Leverson, who was suffering from the effects of an accident.

In the regular order of rotation of Chairman, Dr. Close presided. Calling the meeting to order, he congratulated the members upon a year of most helpful and delightful intercourse, and upon the prospect and promise of the continuance of such relations.

Members have evinced great interest, have attended meetings regularly, and have maintained the warmest fraternal feeling in carrying out the objects of the Union. Valuable papers have been presented, and free and interesting discussions have been had, in which every member has taken part with pleasure and profit, in a series of meetings of a delightfully free and informal character.

After the reading and acceptance of the minutes of the last meeting, Dr. Close read a paper entitled, "Symptoms—A Study" (published in full at page 85 of this number), treating of the nature, varieties, and relative importance of symptoms from the Hahnemannian standpoint. The paper was briefly discussed, and the meeting adjourned to the dining-room, where a repast was served and enjoyed. A vote of thanks was tendered to the host and hostess for their hospitality during the past year, and to Mrs. Close in particular for her labors as Secretary. A half-hour musical programme in the parlor, Mrs. Close at the piano, concluded an enjoyable evening.

